# Supplementary figures and images for: Reprogramming of host energy metabolism mediated by the TNF-iNOS-HIF-1α axis plays a key role in host resistance to Plasmodium infection
Source: eLife. 2026 Jul 27;13:RP97759. doi: 10.7554/eLife.97759 (PMC13405620; doi:10.7554/eLife.97759)

Figure 3.

B.

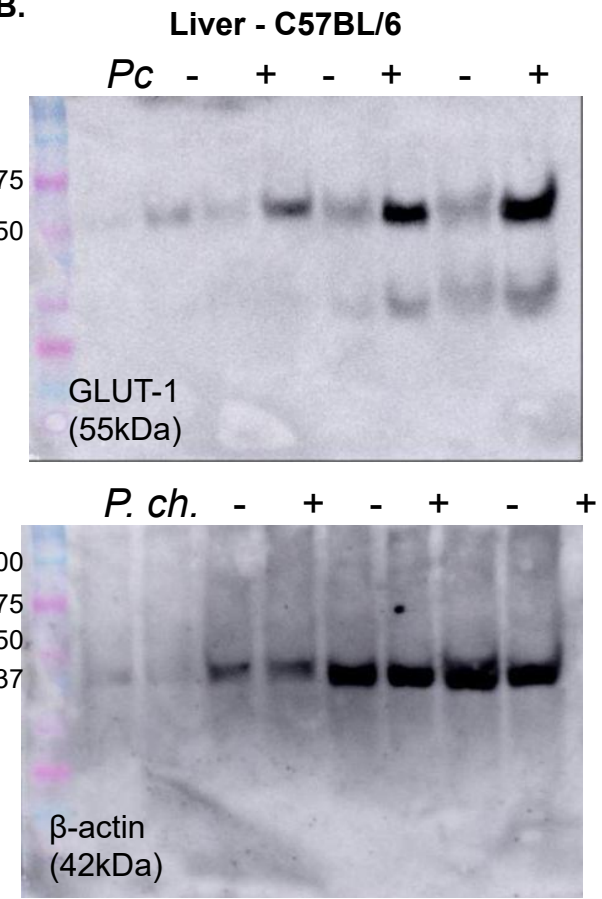

C.

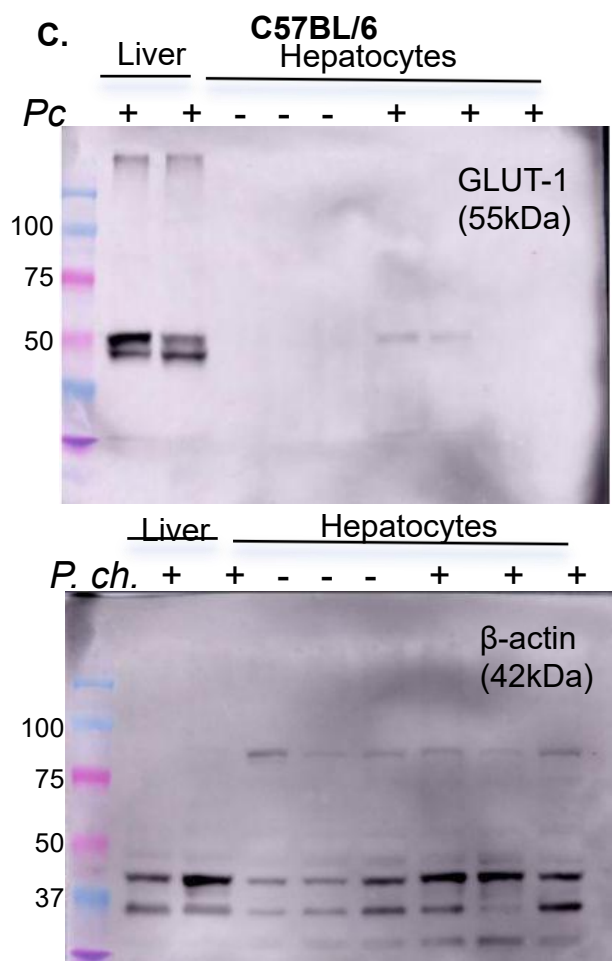

D.

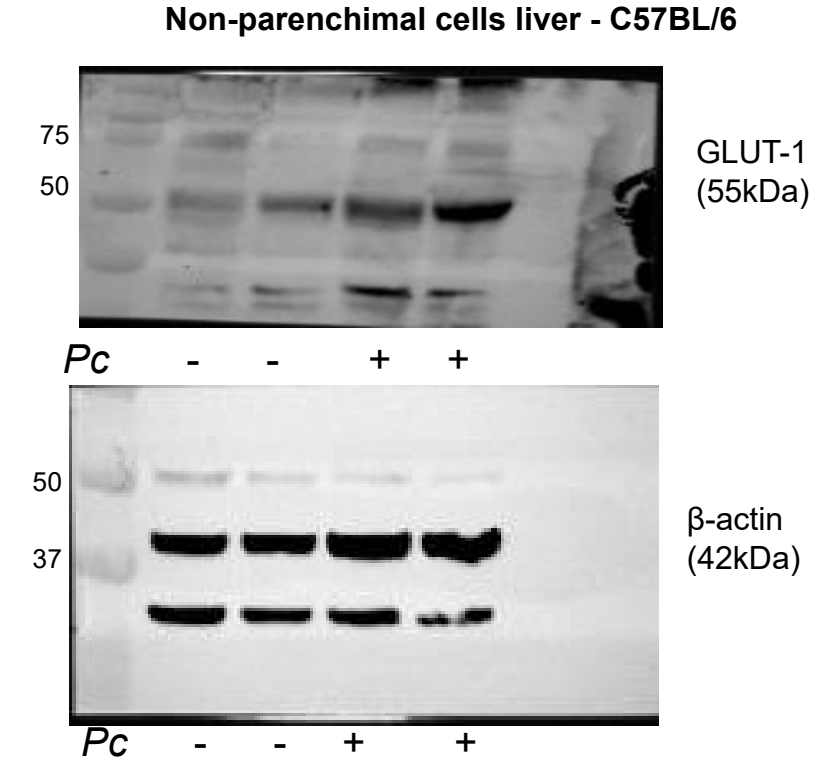

G.

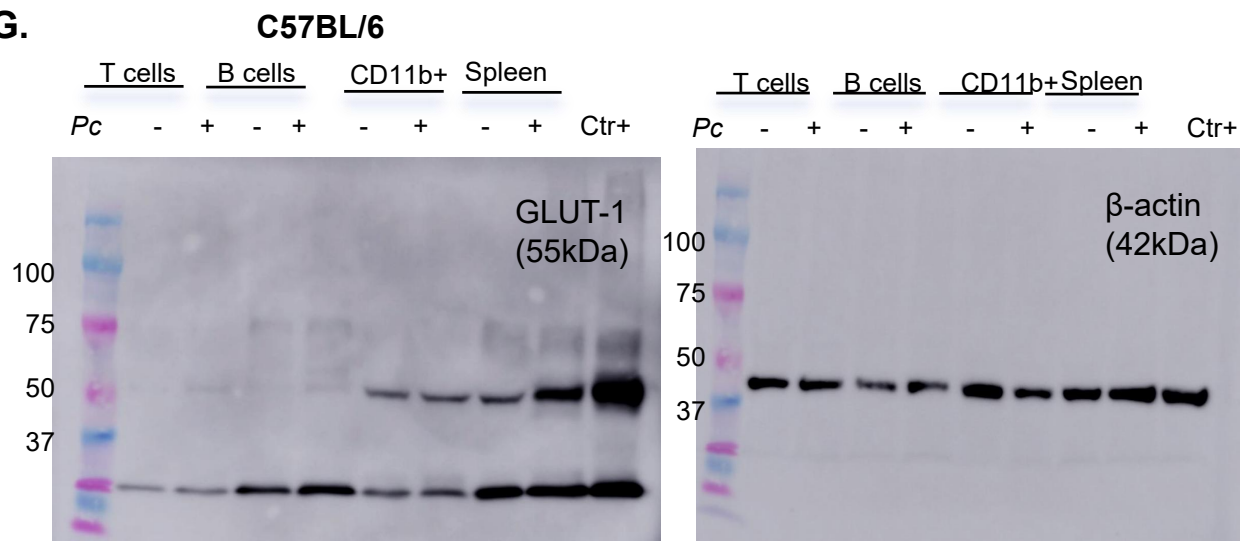

Supplement: Figure 3—source data 1. [file elife-97759-fig3-data1.zip › figures 3 source data1.pdf]

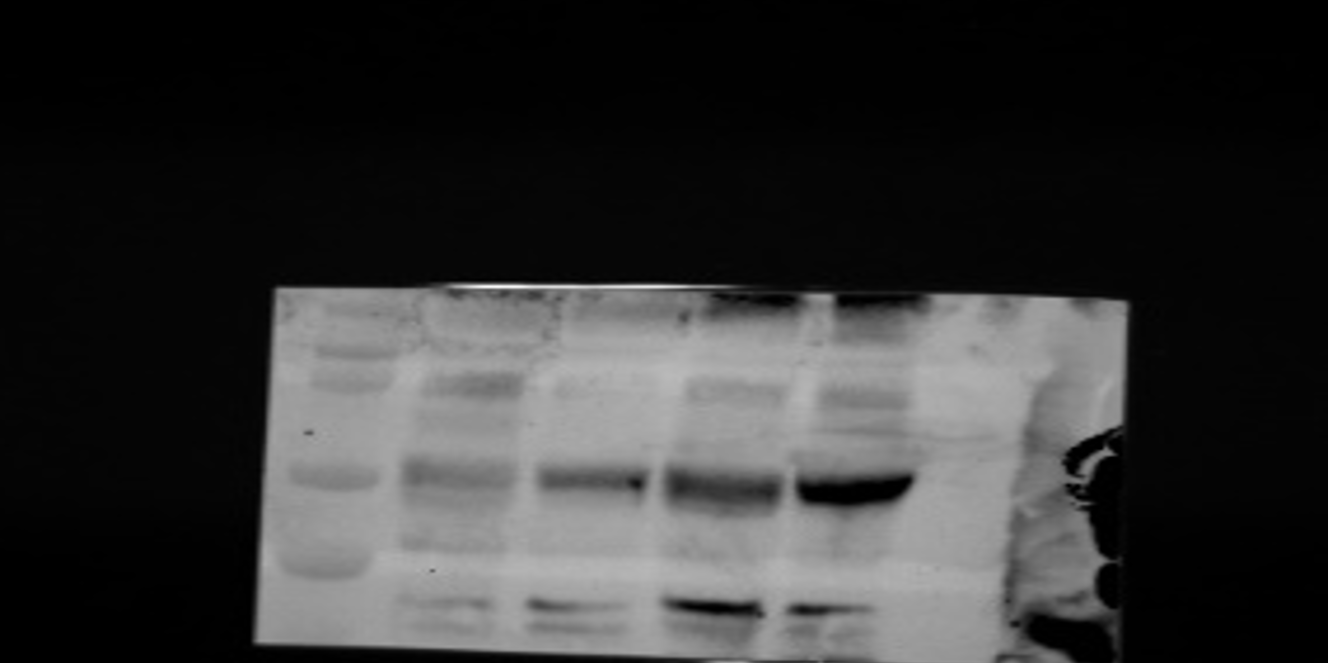

Supplement: Figure 3—source data 2. [file elife-97759-fig3-data2.zip › fig 3d.tif]

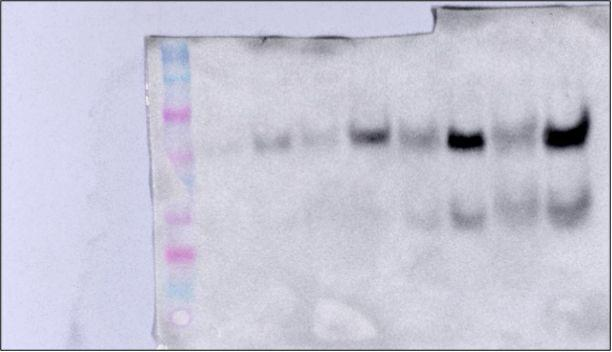

Supplement: Figure 3—source data 2. [file elife-97759-fig3-data2.zip › fig3b.tiff]

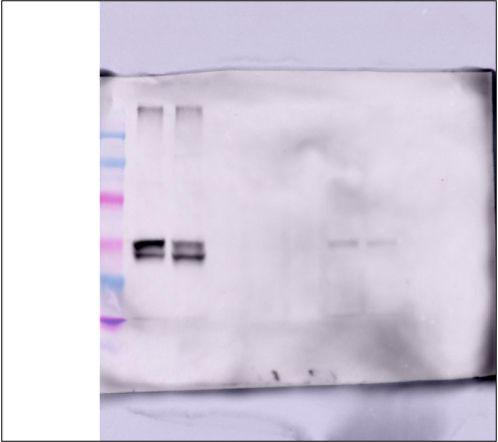

Supplement: Figure 3—source data 2. [file elife-97759-fig3-data2.zip › fig 3c.tiff]

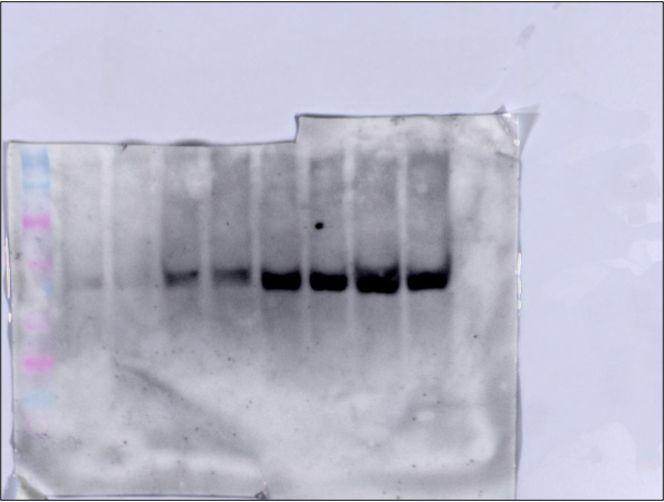

Supplement: Figure 3—source data 2. [file elife-97759-fig3-data2.zip › fig3b (2).tiff]

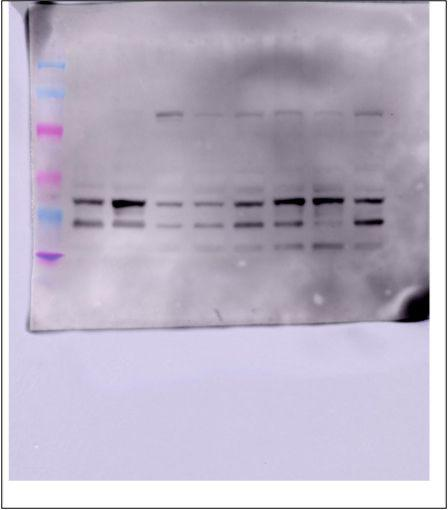

Supplement: Figure 3—source data 2. [file elife-97759-fig3-data2.zip › fig 3c (2).tiff]

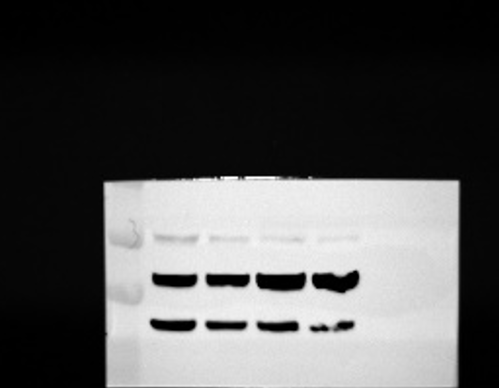

Supplement: Figure 3—source data 2. [file elife-97759-fig3-data2.zip › fig 3d (2).tif]

Figure 4.

K.

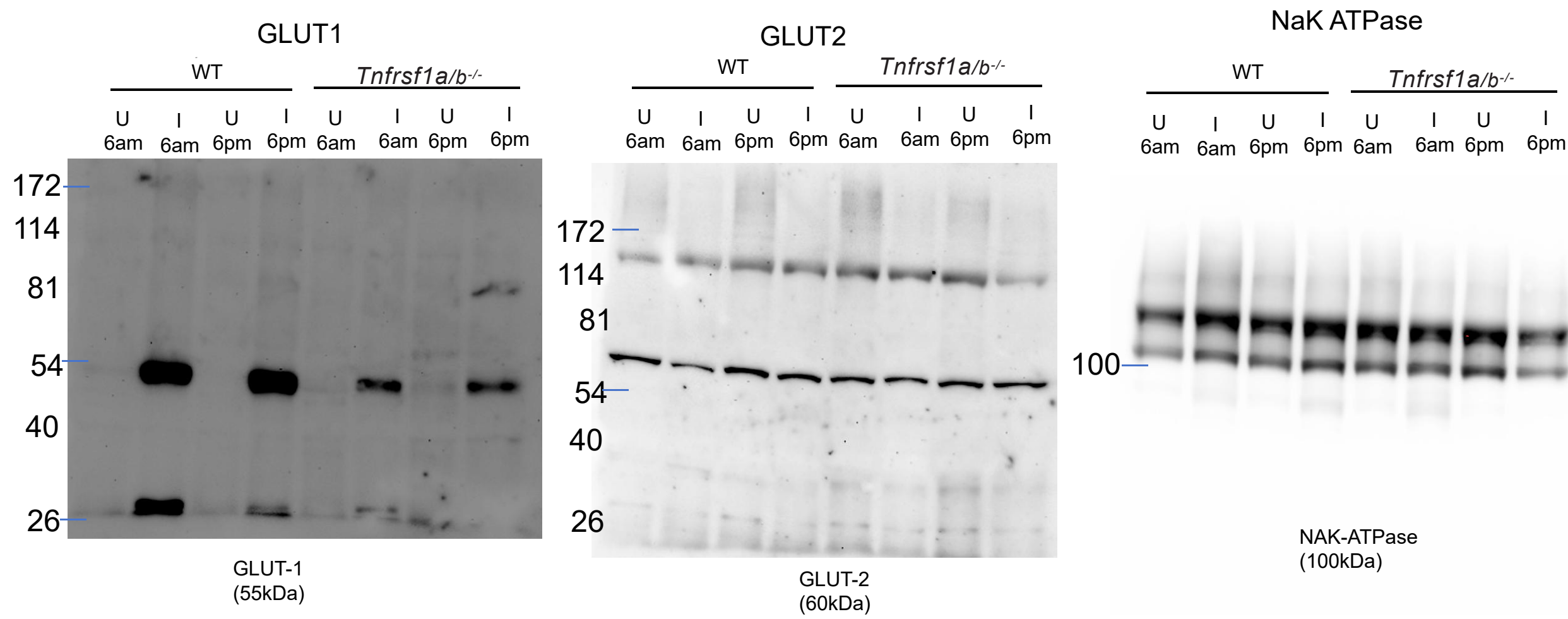

Supplement: Figure 4—source data 1. [file elife-97759-fig4-data1.zip › figures 4 source data1.pdf]

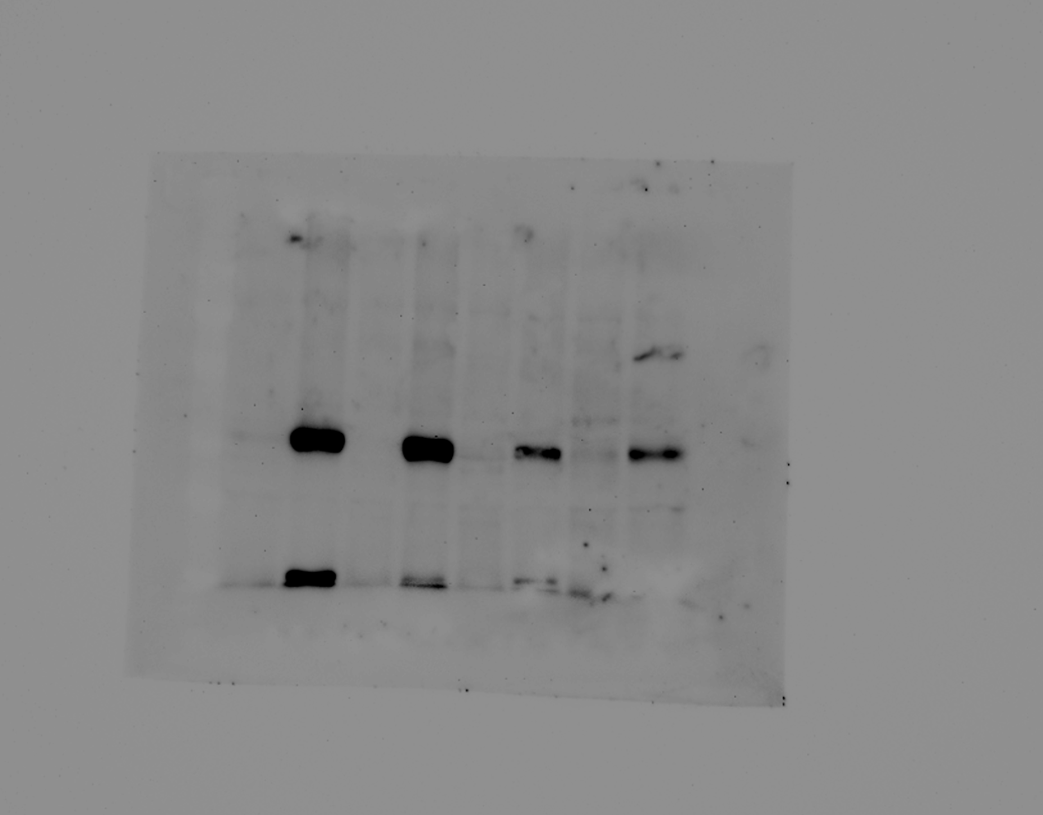

Supplement: Figure 4—source data 2. [file elife-97759-fig4-data2.zip › fig 4k.tif]

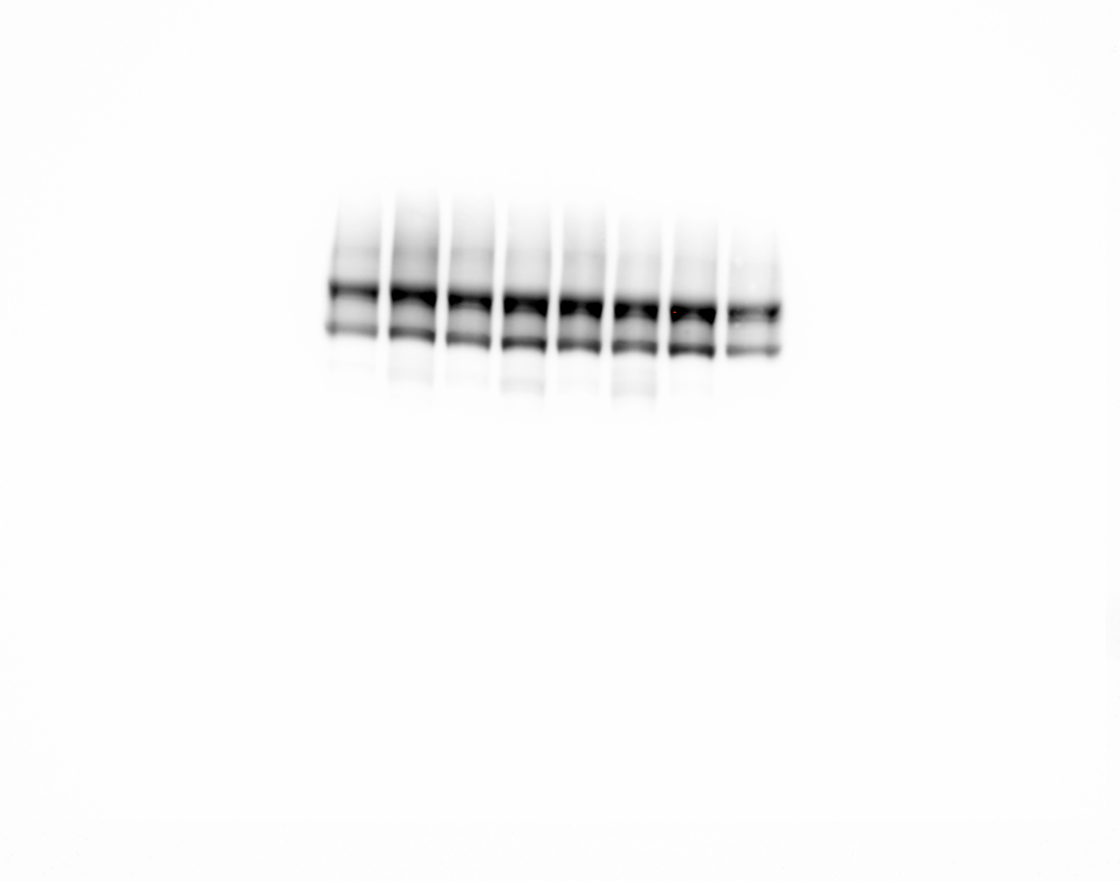

Supplement: Figure 4—source data 2. [file elife-97759-fig4-data2.zip › fig 4k (3).tif]

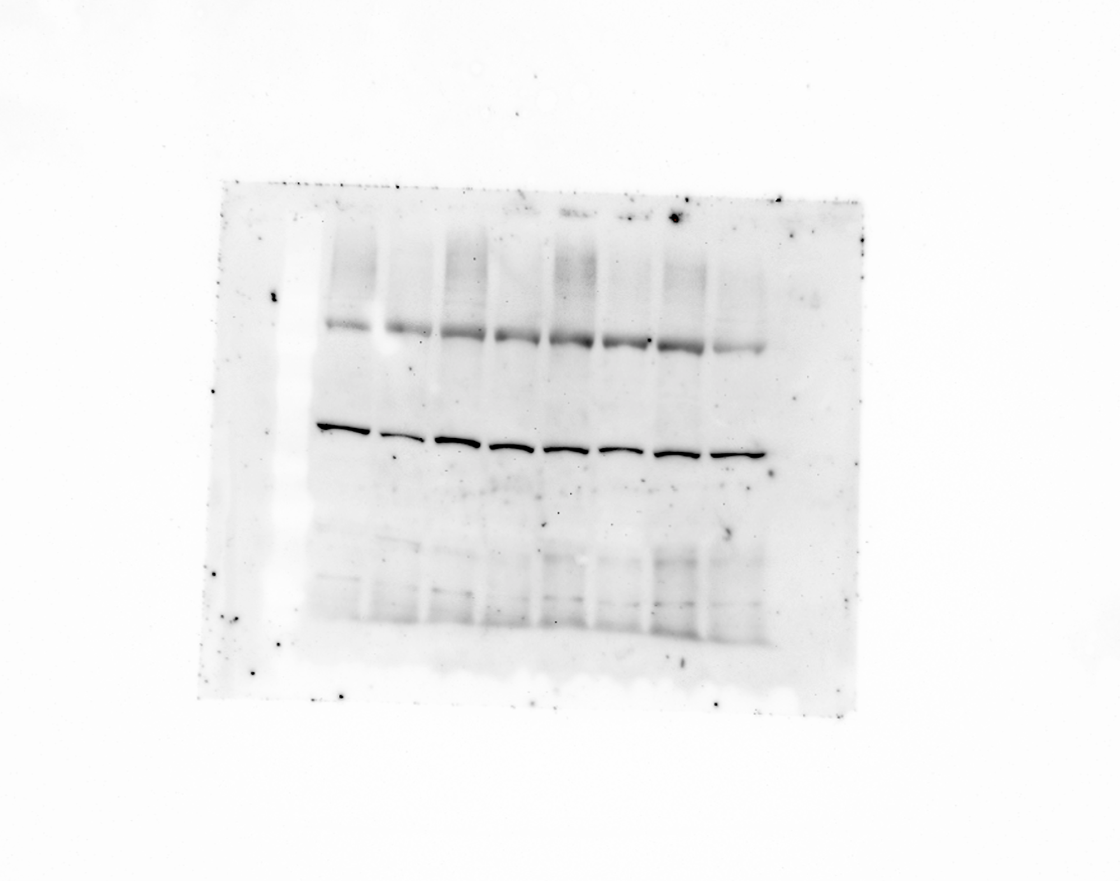

Supplement: Figure 4—source data 2. [file elife-97759-fig4-data2.zip › fig 4k (2).tif]

Figure 5.

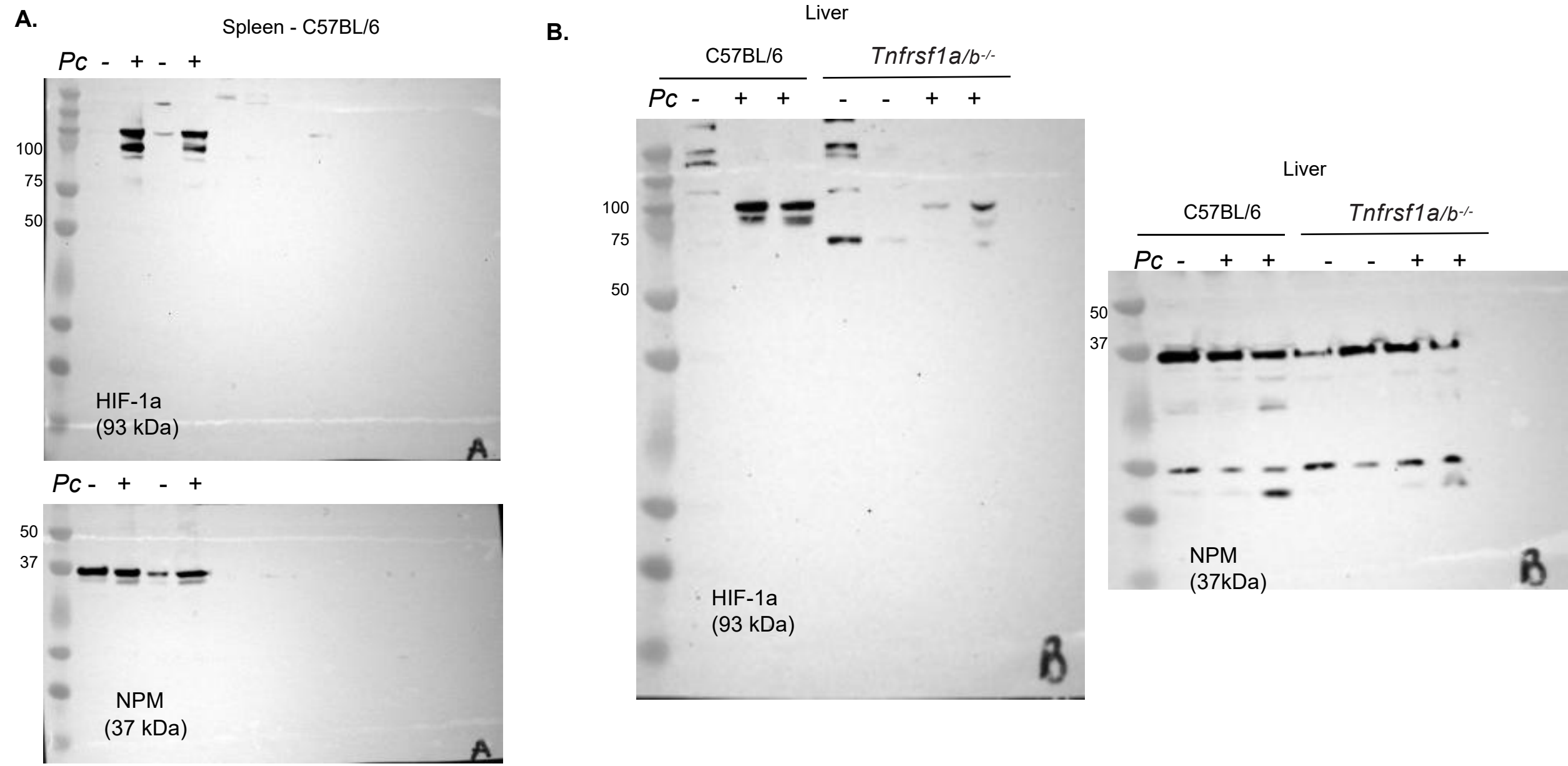

Supplement: Figure 5—source data 1. [file elife-97759-fig5-data1.zip › figures 5 source data1.pdf]

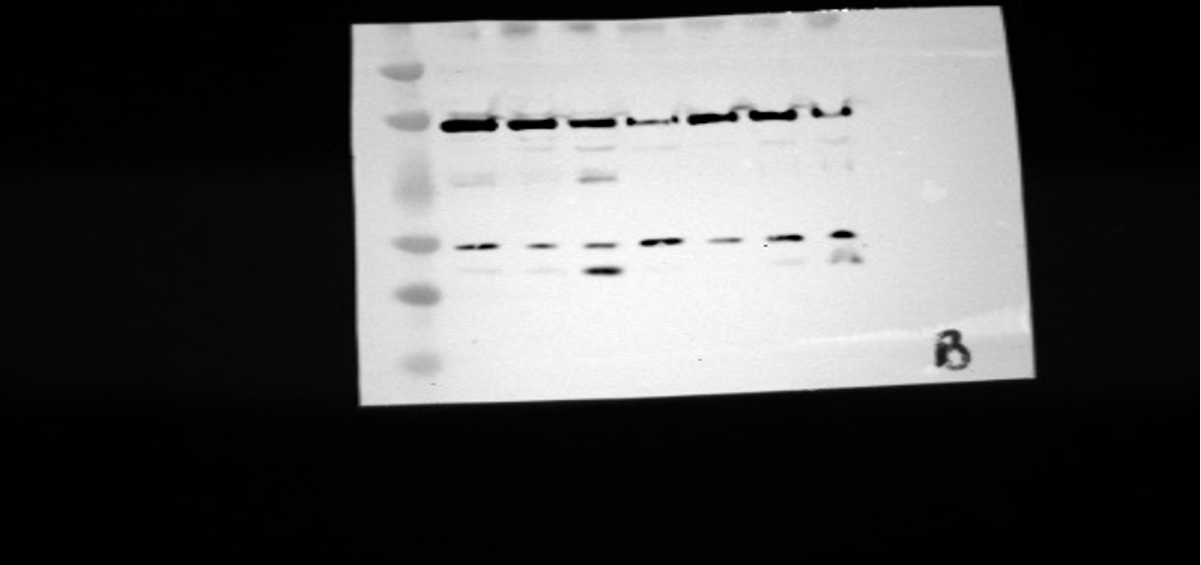

Supplement: Figure 5—source data 2. [file elife-97759-fig5-data2.zip › fig5b.tif]

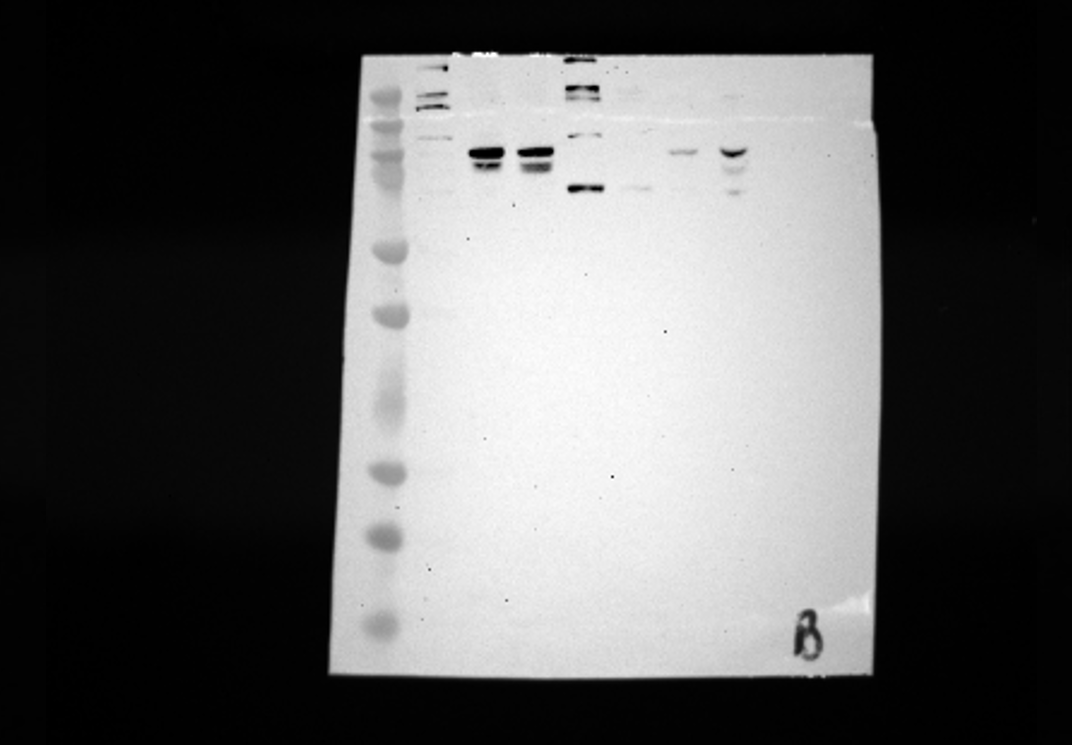

Supplement: Figure 5—source data 2. [file elife-97759-fig5-data2.zip › fig 5b (2).tif]

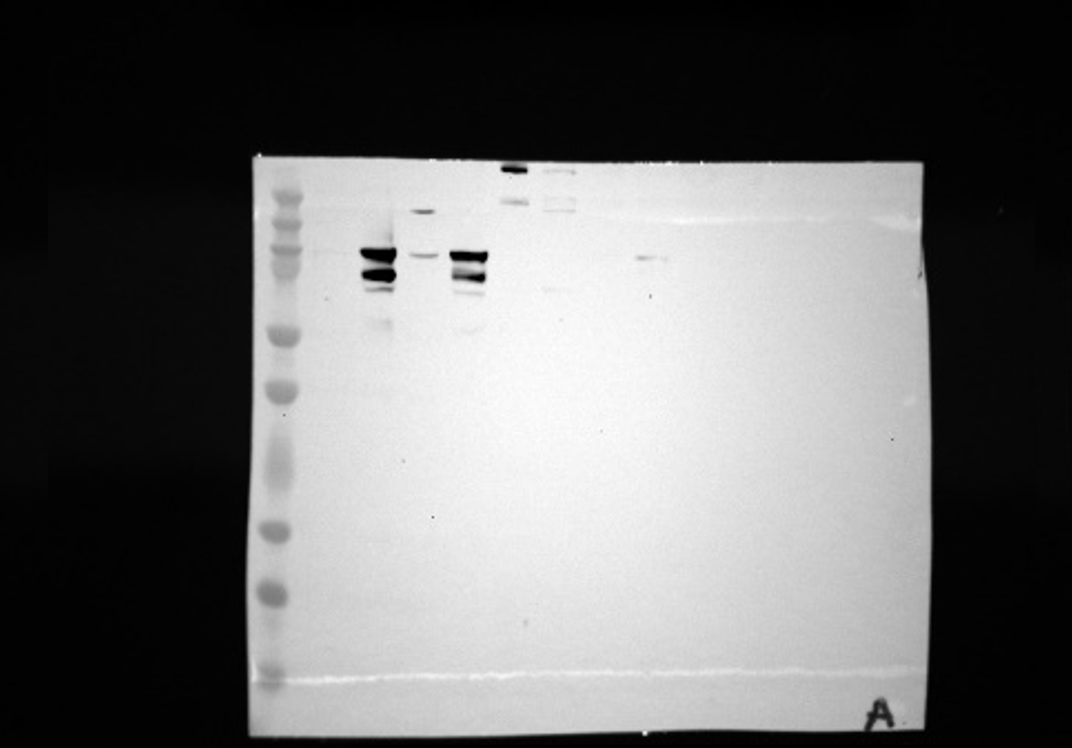

Supplement: Figure 5—source data 2. [file elife-97759-fig5-data2.zip › fig 5a (2).tif]

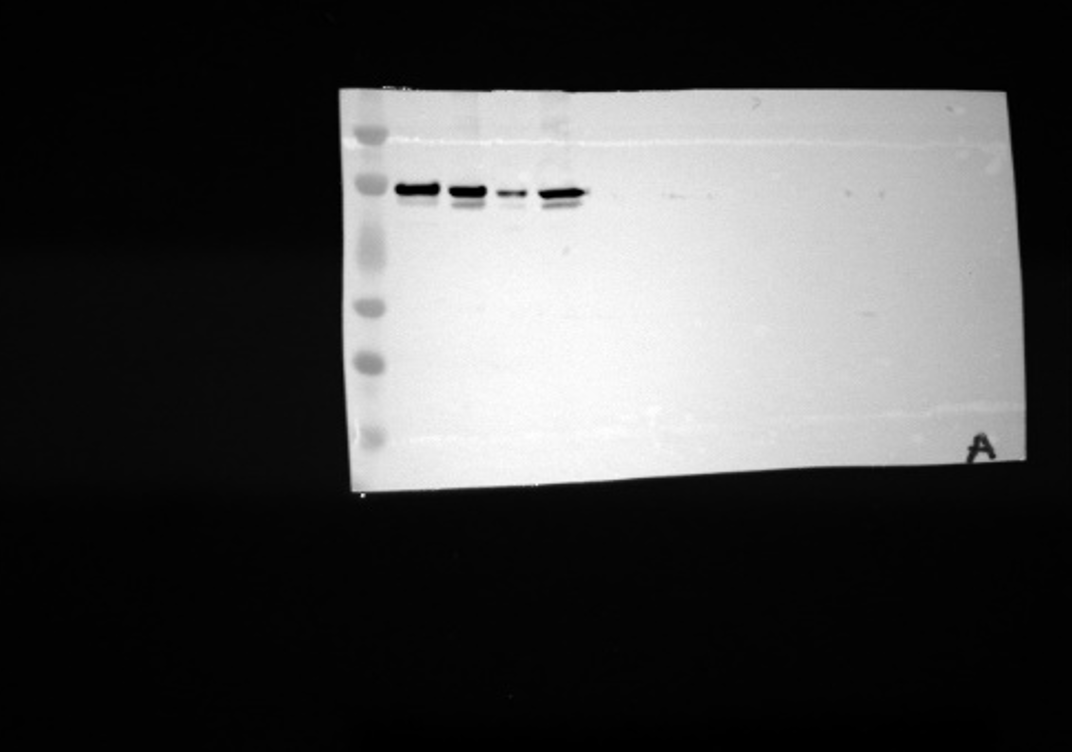

Supplement: Figure 5—source data 2. [file elife-97759-fig5-data2.zip › fig 5a.tif]
