# Supplementary material for: Reprogramming of host energy metabolism mediated by the TNF-iNOS-HIF-1α axis plays a key role in host resistance to Plasmodium infection
Source: eLife. 2026 Jul 27;13:RP97759. doi: 10.7554/eLife.97759 (PMC13405620; doi:10.7554/eLife.97759)
Supplement: Peer review history

# Editor report: eLife Assessment

**DOI:** 10.7554/eLife.97759.4.sa0

**Authors:** Bryan D Bryson

**Affiliations:** Massachusetts Institute of Technology United States

**Keywords:** Important, Solid

This **important** study examines the role of TNF in modulating energy metabolism during parasite infection. The authors perform an elegant set of studies combining genetics, small molecule perturbation, and phenotypic experiments to highlight a role for glycolysis and glucose transport in control of parasitemia. This **solid** work integrates an interesting set of observations that will be of interest to the Plasmodium and pathogenesis communities with an expanded set of experiments.

# Referee report: Reviewer #2 (Public review)

**DOI:** 10.7554/eLife.97759.4.sa1

[Editors' note: this version has been assessed by the Reviewing Editor without further input from the original reviewers.]

Summary:

The premise of the manuscript by Matteucci et al. is interesting and elaborates a mechanism via which TNFa regulates monocyte activation and metabolism to promote murine survival during Plasmodium infection. The authors show that TNF signaling (via an unknown mechanism) induces nitrite synthesis, which (via yet an unknown mechanism), and stabilizes the transcription factor HIF1a. Furthermore, that HIF1a (via an unknown mechanism) increases GLUT1 expression and increases glycolysis in monocytes. The authors demonstrate that this metabolic rewiring towards increased glycolysis in a subset of monocytes is necessary for monocyte activation including cytokine secretion, and parasite control.

Strengths:

The authors provide elegant in vivo experiments to characterize metabolic consequences of Plasmodium infection, and isolate cell populations whose metabolic state is regulated downstream of TNFa. Furthermore, the authors tie together several interesting observations to propose an interesting model.

Weaknesses:

The authors show that TNFa induces GLUT1 in monocytes, but do not show a direct role for GLUT1 or glucose uptake in monocytes in host resistance to infection.

# Author comment: Author response

**DOI:** 10.7554/eLife.97759.4.sa2

**Authors:** Kely Catarine Matteucci, Nathalia PS Leite, Patricia A Assis, Isabella Cristina Hirako, Franciele Pioto, Ogooluwa Ojelabi, Juliana E Toller-Kawahisa, Leonardo Gomes Vaz, Diego Luis Costa, Joao S Silva, José C Alves-Filho, Ricardo Gazzinelli

**Affiliations:** Universidade de São Paulo Ribeirão Preto Brazil; Universidade de São Paulo Ribeirão Preto Brazil; University of Michigan Michigan United States; Fundação Oswaldo Cruz Belo Horizonte Brazil; Fundação Oswaldo Cruz Ribeirão Preto Brazil; University of Massachusetts Chan Medical School Worcester United States; Trinity College Dublin Dublin Ireland; Universidade Federal de Minas Gerais Belo Horizonte Brazil; Universidade de São Paulo São Paulo Brazil; Universidade de São Paulo Ribeirão Preto Brazil; University of Sao Paulo Ribeirao Preto Brazil; University of Massachusetts Chan Medical School Massachusetts United States

The following is the authors’ response to the previous reviews

We thank the reviewers for their careful evaluation and constructive comments throughout the two rounds of revision. We hope that the revisions have satisfactorily addressed all concerns and that the manuscript is now suitable for publication.

This novel contribution highlights the role of this pro-inflammatory factor in the pathogenesis of and resistance to *Plasmodium chabaudi* infection in mice. While aspects of this response have been previously described, this study is the first to link the TNF–iNOS–HIF-1α axis to the in vivo mediation of malaria disease through its involvement in glucose metabolism. Despite well-documented metabolic alterations during malaria, including hypoglycemia and hyperlactatemia, the mechanisms underlying these changes and their relationship to host immune responses remain poorly understood. Addressing this gap is essential for elucidating how metabolic adaptation shapes disease outcomes during *Plasmodium* infection.

In response to the reviewer’s comments, we have revised the Abstract, Introduction, and Discussion to clearly distinguish between:

Previously established mechanisms (TNF–iNOS–HIF-1α–glycolysis axis), and

The novel contribution of our study (its in vivo integration during *Plasmodium* infection and association with host resistance).

**Public Reviews:**

**Reviewer #2 (Public review):**

Summary:

The premise of the manuscript by Matteucci et al. is interesting and elaborates a mechanism via which TNFa regulates monocyte activation and metabolism to promote murine survival during Plasmodium infection. The authors show that TNF signaling (via an unknown mechanism) induces nitrite synthesis, which (via yet an unknown mechanism), and stabilizes the transcription factor HIF1a. Furthermore, that HIF1a (via an unknown mechanism) increases GLUT1 expression and increases glycolysis in monocytes. The authors demonstrate that this metabolic rewiring towards increased glycolysis in a subset of monocytes is necessary for monocyte activation including cytokine secretion, and parasite control.

Strengths:

The authors provide elegant in vivo experiments to characterize metabolic consequences of Plasmodium infection, and isolate cell populations whose metabolic state is regulated downstream of TNFa. Furthermore, the authors tie together several interesting observations to propose an interesting model regarding

Weaknesses:

The main conclusion of this work - that "Reprogramming of host energy metabolism mediated by the TNF-iNOS-HIF1a axis plays a key role in host resistance to Plasmodium infection" is unsubstantiated. The authors show that TNFa induces GLUT1 in monocytes, but never show a direct role for GLUT1 or glucose uptake in monocytes in host resistance to infection (nor the hypoglycemia phenotype they describe).

We thank the reviewer for this important comment and for highlighting the need to clarify the mechanistic link between TNF-driven metabolic rewiring and host resistance to *Plasmodium* infection. As noted in our first revision, our primary objective was to investigate how TNF integrates systemic and cellular metabolic responses during infection in vivo. We demonstrate that glucose uptake is significantly increased in spleen and liver during infection in a partially TNF-dependent manner, and that TNF promotes GLUT1 expression (main glucose transporter in immune cells) and glycolysis specifically in monocytic cells. Importantly, to directly address the role of TNF signaling in myeloid cells, we also observed the same phenotype (higher parasitemia, but absence of hypothermia and hypoglycemia) in mice with conditional deletion of TNF receptor 1 in lysozyme M–expressing cells (TNFR1^ΔLyz2) (Figure 4P–R), thereby validating in a cell-specific context the findings previously observed in mice with global TNFR1 deficiency. Together, these findings support a functional link between TNF signaling in monocytes, induction of GLUT1-dependent glucose metabolism, and the regulation of both systemic metabolic responses and host resistance during experimental malaria.

While we agree that we do not demonstrate a cell-intrinsic role for GLUT1 in monocytes, multiple lines of evidence in our study support the functional relevance of glycolytic metabolism downstream of the TNF–iNOS–HIF-1α axis.

(1) First, we show that *Pc* infection results in a marked increase in glucose uptake in the spleen and liver, but not in skeletal muscle or adipose tissues (Figure 2K), and that this effect is absent in TNFR-/- mice (Figure 2L), indicating a TNF-dependent and tissue-specific metabolic reprogramming. We have also clarified in the Discussion that this process appears to be insulin-independent and likely driven by pro-inflammatory signals.

(2) Second, we show that the TNF–iNOS–HIF-1α axis. induces GLUT1 expression in monocytic cells (Figures 4M, 5D, 6L). This supports a model in which these cells contribute to observed systemic metabolic changes.

(3) Third, we also observed a similar phenotype—characterized by higher parasitemia but absence of hypothermia and hypoglycaemia-in mice with conditional deletion of TNF receptor 1 in lysozyme M–expressing cells (TNFR1^ΔLyz2) (Figure 4P–R), thereby validating in a cell-specific context the findings previously observed in mice with global TNFR1 deficiency. These findings indicate that disruption of glycolysis phenocopies key aspects of the TNF-driven metabolic and immunological response to infection.

(4) Finally, we demonstrate that glycolytic metabolism is functionally relevant for host resistance. Pharmacological inhibition of glycolysis in vivo using 2-DG led to increased parasitemia (Figure 6O), resembling the impaired parasite control observed in HIF-1α^ΔLyz2, TNFR-/-, and iNOS-/- mice. These findings indicate that disruption of glycolysis phenocopies key aspects of the TNF–iNOS–HIF-1α axis deficiency, supporting the conclusion that this pathway is required to sustain glycolytic metabolism and effective parasite control during infection.

About the hypoglycemia phenotype and resistance, our previous study (PMID: 29805094) demonstrates that TNF-driven inflammation regulates systemic glucose metabolism during *Plasmodium chabaudi* infection. We showed that infection-induced hypoglycemia correlates with TNF levels and is associated with changes in parasite development. Specifically, leukocytes primed with IFNγ display increased expression of glucose metabolism and inflammatory genes, and TNFα-induced hypoglycemia is linked to the accumulation of non-proliferative trophozoite forms, whereas parasite replication (schizogony) occurs during host feeding. These findings indicate that blood glucose availability, regulated by TNF, directly influences parasite growth dynamics and infection outcome. Although the cellular mechanisms were not addressed in that study, our current work builds on these findings by identifying the TNF-iNOS–HIF-1α axis as a driver of GLUT1-dependent glycolysis in monocytes, linking systemic metabolic changes to a cell-intrinsic mechanism that contributes to host resistance.

We agree that directly establishing the cell-intrinsic contribution of GLUT1 would require dedicated genetic approaches (e.g., conditional deletion in monocytes), which are beyond the scope of the present study.

Comments on revisions:

The demonstration that the established TNF-iNOS-HIF-1α-glycolysis axis operates in vivo during P. chabaudi infection is valuable and relevant. However, it constitutes contextual validation and must be carefully described as such. This distinction, i.e., "what has already been shown vs. what is new" is not consistently reflected in the framing of the manuscript raising overstatement concerns. This is particularly evident in the abstract and other conclusive statements, where mechanistic novelty is implied, even when the underlying pathways/mechanisms are already known. To improve the manuscript, all sentences that refer to already established findings should be accurately described as such.

For example, the abstract states: "Here, we show that TNF signaling hampers physical activity, food intake, and energy expenditure while enhancing glucose uptake by the liver and spleen as well as controlling parasitemia in *P. chabaudi*-infected mice." In this sentence, the effects of TNF signaling on physical activity, food intake, energy expenditure, glucose metabolism and control of parasitemia are unequivocally established and therefore do not, in themselves, constitute new findings. Feeding behavior, not cell-intrinsic metabolism, may drive glycemic differences.

We thank the reviewer for this comment and for highlighting the importance of distinguishing systemic metabolic effects from cell-intrinsic mechanisms. We have now revised the manuscript to more consistently distinguish between previously established mechanisms and our novel findings, particularly in the Abstract and other summary statements, to avoid any potential overstatement.

We also would like to emphasize that, in both the Introduction and Discussion, we explicitly acknowledge that key components of the TNF–iNOS–HIF-1α–glycolysis axis have been previously described. In the Introduction, we cite studies demonstrating that TNF can induce glucose uptake and metabolic reprogramming in immune cells (refs. 14–17), as well as the role of HIF-1α as a central regulator of glycolysis and inflammation in myeloid cells (refs. 21–28). Similarly, in the Discussion, we detail prior evidence that TNF induces iNOS-derived RNI (refs. 51–54), that RNI stabilizes HIF-1α (ref. 52), and that HIF-1α drives the expression of glycolytic genes including GLUT1 (refs. 55–57). We also cite studies showing that TNF contributes to parasite control and glucose metabolism in malaria (refs. 58–61).

Importantly, while these pathways have been described in other contexts, their integration and functional relevance in vivo during *Plasmodium* infection, particularly in the context of host systemic metabolism and monocytic cell function, have not been previously demonstrated. Our study addresses this gap by showing that this axis operates during *P. chabaudi* infection and links inflammatory signaling to both cellular metabolic reprogramming and organismal metabolic changes.

Specifically, we demonstrate that TNF signaling drives increased glucose uptake in spleen and liver in a tissue-specific manner, promotes GLUT1 expression and glycolysis in monocytic cells, and that disruption of this axis (genetically or pharmacologically via glycolysis inhibition) impairs parasite control. In addition, we provide evidence connecting these cellular processes to systemic metabolic alterations, including hypoglycemia.

The authors propose that TNF signaling leads to GLUT1 upregulation (in inflammatory monocytes, MO-DCs, and within the liver and spleen) during Plasmodium infection, and that this results in increased glucose uptake contributing to systemic hypoglycemia. While this is an intriguing hypothesis, we urge the authors to consider an alternative explanation that, at present, is not adequately ruled out. Given that glycemia serves as a central functional readout in the manuscript, this distinction is essential to clarify.

The observed regulation of glycemia is likely not a direct consequence of increased glucose uptake by immune cells or by tissues but may instead reflect broader differences in disease severity across genotypes. The iNOS KO, TNFR KO, and HIF-1ΔLyz2 mice likely experience a dampened inflammatory response, which would blunt infection-induced anorexia and help preserve overall metabolic homeostasis. This alternate interpretation is supported by the authors' metabolic cage data showing increased physical activity in TNFR KO mice and the elevated food intake shown in Figure 2B.

We thank the reviewer for this important point regarding the potential contribution of feeding behavior and systemic energy balance to the observed metabolic phenotypes. In fact, this possibility has been explicitly already incorporated into the revised manuscript. Also, we have revised the Discussion to explicitly state that the hypoglycemia observed during infection likely reflects both systemic changes in energy balance and TNF-driven metabolic reprogramming in immune cells, rather than a single isolated mechanism. Specifically, we have had already added the following statement to the Discussion:

“Although restored physical activity, food consumption and energy expenditure in knockout mice may contribute to the observed systemic metabolic parameters by altering energy balance, these effects are not mutually exclusive with the TNF-driven, cell-intrinsic metabolic mechanisms described here”.

In addition, we note that under naive conditions, we did not observe differences between genotypes in physical activity, food intake, energy expenditure, respiratory exchange ratio, or glycemia. These findings support that baseline metabolic parameters are comparable and that the differences observed during infection arise in the context of TNF-dependent inflammatory responses. During infection, although TNFR-deficient mice display increased food intake and activity, these differences arise in the context of altered inflammatory signaling. Therefore, rather than being mutually exclusive, behavioral and metabolic changes are likely coordinated downstream of TNF signaling.

Furthermore, our data using pharmacological inhibition of glycolysis (2-deoxy-D-glucose) demonstrate that disruption of glycolytic metabolism results in increased parasitemia and reduced lactate levels, recapitulating key aspects of the phenotype observed in TNFR-/-, iNOS-/-, and HIF-1αΔLyz2 mice. This supports a functional role for glycolytic metabolism in host response, beyond differences in feeding behavior.

Since anorexia and energy expenditure are tightly coupled to the inflammatory milieu, it is plausible that these behavioral and systemic differences-not monocyte nor tissue GLUT1 expression per se-are the primary contributors to the observed glycemic patterns. To support their current interpretation, the authors should perform a pair-feeding experiment in which (at least) TNFR KO mice are restricted to the same food intake as infected WT controls. This would help disentangle whether differences in glycemia truly reflect immune-driven metabolic rewiring or are secondary to differences in caloric intake.

We thank the reviewer for this suggestion. We agree that pair-feeding experiments would provide an additional layer of control to isolate the contribution of caloric intake. However, we note that:

(1) Baseline metabolic equivalence in naive animals argues against intrinsic differences in energy balance.

(2) The observed phenotypes occur in the context of infection-driven inflammation, where anorexia is itself a TNF-dependent host response.

(3) Our data support a model in which behavioral changes and metabolic rewiring are integrated components of the host response rather than independent variables.

Importantly, our data already support a role for TNF-driven metabolic rewiring beyond feeding behavior, as inhibition of glycolysis with 2-deoxy-D-glucose recapitulates the impaired parasite control observed in genetic models. In addition, as discussed in the manuscript, systemic factors such as food intake are not mutually exclusive with cell-intrinsic metabolic mechanisms.

We therefore consider that pair-feeding experiments are beyond the scope of the present study.

The contribution of monocyte-specific glucose metabolism to host resistance remains unresolved.

We appreciate the authors' effort to address the mechanistic role of glycolysis in host resistance using in vivo 2-deoxyglucose (2DG) treatment. However, I would like to point out that while this experiment is informative, it does not fully resolve the specific concern raised regarding the cell-intrinsic role of TNF-induced glycolysis in monocytes. 2DG acts systemically, inhibiting glycolysis across a wide range of cell types-including hepatocytes, endothelial cells, lymphocytes, and myeloid populations. Therefore, the observed increase in parasitemia following 2DG treatment may reflect the broad importance of glycolysis for host defense, or alternatively, may result from elevated circulating glucose levels induced by 2DG (PMID: 35841892), which could enhance parasite growth by increasing nutrient availability. Therefore, this experiment does not allow for a specific conclusion about the requirement for TNF-driven metabolic reprogramming in monocytes.

We thank the reviewer for this comment regarding the interpretation of the 2-deoxyglucose (2DG) experiments. We agree that systemic 2DG treatment does not allow cell-specific conclusions, as it broadly inhibits glycolysis across multiple cell types. Accordingly, these data are interpreted as supporting a role for glycolysis in host defense at the organismal level, rather than as direct evidence for a monocyte-intrinsic requirement of TNF-driven metabolic reprogramming.

At the same time, our study includes cell-specific analyses that support the engagement of this pathway in myeloid populations. In particular, we observe increased GLUT1 expression in CD11b^+^ cells within both the liver and spleen during infection, with marked upregulation in monocyte-derived dendritic cells (MODCs). Importantly, this induction is not observed in the corresponding knockout models, supporting the idea that TNF signaling is required for this metabolic adaptation in these cells in vivo. Consistent with this, we validated that both parasitemia and systemic glucose levels in TNFR1^ΔLyz2 mice phenocopy those observed in TNFR-deficient animals, reinforcing the contribution of myeloid TNF signaling to the metabolic and disease outcomes.

In addition, our in vitro data demonstrate increased GLUT1 expression in WT monocytes but not in cells lacking components of the TNF–iNOS–HIF-1α axis, further supporting a pathway-specific effect. Given that GLUT1 is the primary glucose transporter in immune cells, these combined in vivo and in vitro findings, together with the 2DG experiments, provide strong evidence supporting our proposed model.

We agree that directly establishing a monocyte-intrinsic role would require targeted genetic approaches, which are beyond the scope of the present study.
